# The Use of Alcohol from a Medical Standpoint
1Read as the introduction to a discussion at the meeting of the Bristol Medico-Chirurgical Society on January 8th, 1896. A report of the discussion will be found under "Meetings of Societies."


**Published:** 1896-03

**Authors:** John Dacre

**Affiliations:** Surgeon to the Bristol Hospital for Sick Children and Women


### THE USE OF ALCOHOL FROM A MEDICAL STANDPOINT.[1]

BY

John Dacre, M.R.C.S. Eng., L.R.C.P. Lond.,

*Surgeon to the Bristol Hospital for Sick Children and Women.*

---

The occasional opportunity of rubbing shoulders over well-worn paths can only be for mutual good. We are apt to lapse into the fatal insecurity of imagining that our views on a certain subject are fully formed and crystallised and that our knowledge is sufficiently complete; and it is only by renewing our acquaintance with old facts that we are brought to our knees to realise how much we have forgotten, and, in fact, how much there is that we never knew before.

In discussing the medical uses of alcohol, first and foremost we must have a basis on which to build our assertions, and it is therefore necessary that its physiological action must be first considered.

Lauder Brunton says that the study of the physiological action of alcohol "is undoubtedly a difficult task, and one which we cannot at present hope to accomplish perfectly. All that we can do is to take the facts we find and arrange them to the best of our ability, trusting to future research for information on those points of which we are now ignorant. In doing this we must bear in mind that alcohol has a threefold action. 1st. Its local action on the skin or mucous membrane with which it comes in contact. 2nd. Its reflex action on other organs, through the sensory nerves of the skin or mucous membranes. 3rd. Its action on the brain, spinal cord, and other organs to which it is conveyed by the blood. . . . Thus, if we moisten the skin with pure alcohol . . . and allow it to dry spontaneously, a decided sensation of cold will be produced. . . . This cooling action is due simply to the

[1] Read as the introduction to a discussion at the meeting of the Bristol Medico-Chirurgical Society on January 8th, 1896. A report of the discussion will be found under "Meetings of Societies."

volatility of alcohol, which during its evaporation abstracts heat from the skin and cools it down. If pure it evaporates quickly and produces much cold, but if mixed with much water the evaporation . . . is too slow to produce any marked result. . . . If, instead of allowing it to evaporate spontaneously, we prevent evaporation altogether by covering the moistened skin with gutta percha tissue, instead of coolness we get a burning feeling. . . . We have got rid of the action which alcohol owes to its volatility, and we have brought into play another which it owes to its chemical properties. So long as it could evaporate readily it acted almost entirely on the epidermis, but when evaporation is prevented it soaks through the epithelium and acts on the vascular tissues beneath. This is better seen if, instead of applying the alcohol to the skin, where the epidermis presents a considerable resistance to its passage, we put it into the mouth, where the thinner epithelium offers less obstruction. Almost immediately after its introduction we experience a burning sensation, which increases for a little while and then gradually diminishes. . . . The mucous membrane changes its character and becomes whiter, more opaque, and somewhat corrugated. . . . Both the sensation of burning, and the visible alteration in the mucous membrane, are due to the action of the alcohol upon the tissues."[1] The warmth is produced by the increase of the local circulation, and the whiteness to the coagulation of albumen. This coagulation is more or less lasting according as the alcohol has been acting for a greater or smaller length of time. "The simple experiment of putting a little brandy into the mouth is instructive not only by showing us the local changes which alcohol produces in the mucous membrane, but by reminding us of the second kind of action which alcohol exerts, viz. reflexly through the nervous system. At the same time that the burning sensation is felt, the saliva begins to flow copiously into the mouth. The alcohol has not come into contact with the salivary glands at all, but through the sensory nerves of the mouth it has acted on the nervous centres, and through them upon the vessels and secreting cells of the gland."[2]

[1] *On Disorders of Digestion*, 1893, pp. 141–2. [2] *Op. cit.*, p. 143.

If, instead of allowing the alcohol to linger in the mouth, we swallow it, the same sensation of warmth is felt along the course of the œsophagus and in the stomach as well. The action of alcohol upon the stomach, both locally and by reflex action through its nerve-supply, is most important. And it is here that the important experiments made by Dr. Beaumont upon Alexis St. Martin are so valuable. He found, by observing the mucous membrane of the stomach through the fistula, that the introduction of a little alcohol caused a dilatation of the local blood-vessels, with a corresponding increase in the rosy tint of the surface. The glands began to secrete copiously, beads of gastric juice standing out upon the surface, eventually coalescing and running together in a stream. With this increase in the flow of gastric juice there was a corresponding desire for food ; in fact, the experiment had produced an appetite. The movements of the stomach were increased, and we can readily understand how this would assist the mixture of food with the gastric secretion and accelerate digestion, at the same time assisting in the expulsion of gases and the passage of food into the duodenum.

By gradually increasing the quantity of alcohol in the stomach it is possible to produce quite a contrary effect : the rosy tint gives way to pallor, the mucous glands are roused to greater activity, mucus coats the surface, and eventually nausea and vomiting are produced.

We now come to the reflex actions produced by alcohol in the stomach. If a poisonous dose of alcohol be swallowed, the individual falls immediately into a state of unconsciousness from shock; or, as Sir Benjamin Brodie explained it, the alcohol has acted upon the heart and vessels, much in the same way as a blow upon the epigastrium. "He found that a somewhat small dose of alcohol injected into the stomach of a cat knocked it down senseless, and in this condition it remained for about eight minutes. Then it recovered and walked about. There was no time here for the elimination or for the destruction of the alcohol in the system, and consequently this effect which passed off so rapidly could not be due to the presence of the drug in the blood, and must be attributed to its action upon the stomach before absorption. But when larger quantities were given the animal did not recover in this short time. Then the primary shock lasted so long that before it passed off absorption had time to take place, the alcohol having found its way into the blood was carried by it to the nervous centres, acted upon them, and the shock passed into alcoholic coma. While large doses thus paralyze the heart more or less completely, moderate doses stimulate it to act with increased rapidity, and at the same time with increased force."[1] This reflex action of alcohol upon the heart through the stomach is a most important one, for it is by this action that circulation is restored when, from syncope or other cause, it has nearly ceased.

We now proceed one step further, and trace the effects of alcohol upon the body after its absorption into the blood. We will first consider its action upon the blood itself, and then upon the tissues, directly or indirectly, as it is conveyed to them by the circulation.

It has been found by experiment that strong alcohol injected into the veins causes a coagulation of the blood; it is therefore only with dilute alcohol that we have to deal, as the effect of a local coagulation in the veins of the stomach or intestines would be to effectually bar its further progress. This diluted alcohol first comes into contact with the corpuscles of the blood, and it has been found that the amœboid movements of the white corpuscles are first increased and then diminished; but whether this effect or any other has any practical import or not has not yet been determined. Its chemical action upon the red corpuscles has, however, been carefully determined. George Harley and Schmiedeberg have found that it lessens their power of giving off oxygen.[2] Dujardin-Beaumetz, as the result of his own experiments, says: "In the blood you bring together two bodies, one alcohol, eager for oxygen, the other hæmoglobin, ready to give up that oxygen under the feeblest influence, even that of an inert gas, and you imagine that no exchange takes place between these two bodies. Such changes *do* take place; alcohol, under the influence of hæmoglobin, is transformed into acetic acid. A portion of the alcohol ingested undergoes combustion at the expense of the

[1] Lauder Brunton, *op. cit.*, p. 149. [2] See Lauder Brunton, *op. cit.*, p. 150.

oxygen of the hæmoglobin of the blood globules. When alcohol is introduced into the economy in non-intoxicating doses, it is in part oxidised, and transformed, first, into acetic acid, then into alkaline acetates, and finally into carbonates."[1]

That alcohol is thus oxidised we may safely assume as an established fact which rests upon a basis of most careful and oft-repeated experiment. Moreover, by observation upon the elimination of alcohol from the body we have corroborative evidence of these statements. Baudot found that only a small fraction of alcohol ingested was excreted by the kidneys. Anstie, Thudichum, Dupré, and Schulinus also confirm the fact that only a fractional portion is eliminated. Lallemand, Perrin, Duroy, and Subbotin, on the other hand, published experiments showing that a goodly proportion was actually excreted from the body; but they used very large doses, more, in fact, than could be oxidized in the time. If we are right in accepting this fact of oxidation, we must admit the title of alcohol to be ranked as *a food.* If it be oxidized it will supply energy for muscular exertion or for maintaining animal heat, or for both. Whilst if it be excreted unchanged, it can only be classed with substances which, after acting on the nervous and muscular systems whilst circulating in the blood, pass out after a while through the eliminatory organs. Moreover, Dr. Hammond, in his *Physiological Researches*, says that when he himself took an insufficient diet and was daily losing weight, the addition of alcohol not only prevented this loss of weight but converted it into actual gain. Dr. Anstie, in his work on *Stimulants and Narcotics*, has collected a number of cases in which persons have lived for a considerable time upon alcohol alone, or along with a quantity of food so small as to have been utterly inadequate without it.[2]

We will now carry this blood along with the circulation to the tissues of the body. It is laden with this energy-giving oxidized alcohol, but, at the same time, the hæmoglobin has been robbed of oxygen, and consequently has less to hand over in the process of tissue-combustion: the process of oxidation in

[1] Burney Yeo, *Food in Health and Disease*, 1889, p. 121.

[2] See Lauder Brunton, *op. cit.*, pp. 152–3.

the tissues must necessarily be lessened. Now, although a certain amount of heat has been generated by the previous process in the blood itself, we now have to face the fact that less animal heat must of necessity be formed in the usual manner, and consequently, instead of alcohol actually adding to the temperature of the body, we may, by decreasing the normal chemical activity at the terminals, actually counterbalance this increase with a diminution of temperature as determined by the thermometer. This diminished oxidation of the tissues is, however, not the only method by which alcohol lowers the temperature of the body, for, as we shall presently see, the inhibition of vaso-motor influence in the skin by alcohol leads naturally to dilatation of the superficial capillaries, and with this an irradiation of heat into the air. And in speaking of this action of alcohol upon the heart and vessels, I cannot refrain from giving *verbatim* an excellent word-picture of Lauder Brunton. He says: "One of the best possible opportunities of studying the earlier and slighter effects of alcohol is afforded by a public dinner. If we look at our own hands or those of our neighbours before going in, especially if the ante-room is somewhat cold, we may find them somewhat pinched-looking; the colour somewhat dusky and distributed in patches instead of being uniform; the veins very thin, almost like threads. They are of a somewhat dark blue colour, and on emptying them by pressure they fill very slowly, showing that the circulation is languid. After a few glasses of wine, however, their appearance begins to change. The hands now assume a uniform rosy tint, showing that the capillaries are now dilated and filled with bright arterial instead of dark venous blood; the veins swell up, become prominent, of a light blue colour almost like arteries, and when emptied by pressure fill rapidly, showing that the circulation has become very quick, and that they, like the capillaries, are now filled with blood which is tolerably bright, if not quite arterial, instead of the dark blood they previously contained. The hands entirely lose their shrunken look, little wrinkles in the skin disappear, indeed the hands become larger than usual, or, as my neighbour at a dinner-table once expressed it, they become 'podgy,' from the amount of blood and intercellular fluid in the vessels and tissues; and rings previously loose become almost too tight. This dilatation of vessels, so readily seen in the hands, is not confined to them, but occurs generally throughout the body. The warm blood pouring from the interior of the chest and abdomen over the surface imparts to it a pleasing glow, and a most agreeable feeling of comfort pervades the whole frame. The face shares the general flush, and the pulsation of the temporal arteries not unfrequently becomes easily visible. The current of blood throughout the body is more rapid than before, and this rapidity does not depend simply on the dilated vessels offering less resistance to the current of the blood. No! the alcohol has stimulated the accelerating nerves of the heart, which cause it to pulsate not only more rapidly but more powerfully. . . . Here we have one at least of the most important conditions for the nutrition of all the tissues. The slight diminution in the oxidizing power of blood which alcohol occasions is many times over compensated by the amplitude of its current, and, as this is flowing rapidly through the tissues, bringing them new food and carrying off their waste products, is it any wonder that they work with more than usual vigour? The muscles acquire new strength; the work which previously fatigued them is done with ease; the mental faculties become much more acute, and new ones, previously unsuspected, may even appear. The merchant will be able to drive a harder bargain, the student will solve the problem which previously baffled him, the man who tries with difficulty to speak in a foreign language finds his stammering disappear and his tongue run on with ease, the melancholy man may sing a merry ditty, and the sedate man, whom no one would ever have suspected of such a thing, may succeed in making an excellent joke. Provided the liquor has been good, or, in other words, provided the alcohol employed has been free from all injurious admixture, all these effects, I believe, may be produced, and may pass away without any bad effects."[1]

This little sketch shows us in the most graphic manner the action of a reasonable dose of alcohol upon the heart and circulation generally. The whole vascular system is excited to

[1] *Op. cit.*, pp. 153–5.

more vigorous action, the circulation is accelerated, and the dilatation of superficial vessels gives rise to a subjective feeling of warmth: the brain and nervous system, invigorated with a larger supply of blood, act with greater alacrity, and the individual experiences a sensation of exhilaration and general well-being. Following upon these sensations there comes a reaction, the magnitude of which depends upon the individual, characterised by lassitude and drowsiness, which generally lasts longer than the previous state of excitement. This exhilarating effect upon the brain is in the main due to the invigorated circulation; but if the alcohol be pushed beyond a reasonable limit, we have to reckon with its direct action upon the nervous tissues themselves. There is first of all an inhibitory effect upon the highest centres—the brain acts unfettered by the control of judgment; the individual is unmasked and betrays his true colours; passions and emotions which before were in the background now appear on the surface; and as the motor centres of brain and spinal cord are one by one affected, the picture of alcoholic intoxication is completed.

And now having refurbished our memory, let us see how far these physiological facts correspond with clinical experience. And here it may be as well to remind ourselves that the physiological action of a drug, as determined by experiment, is by no means always commensurate with its therapeutic value; and alcohol is no exception to this. A guinea-pig may be the same as a man to the physiologist, but a cow will not do instead of a countess for *our* purpose. Or, as Moxon[1] puts it: To a physiologist a Queen's Counsel and a potman are alike. He will dissect and decompose the one as easily as the other, and tell both their oxidations up in foot-pounds; but he overlooks the unseen differences which evade scalpel and retort, and which are the very order and stability of human life.

Putting this factor of idiosyncrasy quite on one side, we must always remember that the action of a drug on tissues in health may be quite different in acting upon those tissues when the subject of disease; and whilst admitting that the scientific

[1] *The Alcohol Question.* By Paget, Brunton, and others. Second Edition. [N.D.], p. 98.

3

basis is the only sound one on which to practise our art, still we must admit that our value as practitioners is heightened when we add to that knowledge the light of experience. For instance, if alcohol is to accelerate the heart's action, to hurry along the pulse whenever it is given, we ought never to give it in a case of fever; and yet we know full well this is not so. In cases of pyrexia of asthenic type we prescribe alcohol freely, with every hope of slowing the heart's action, and if this hope is not realised we do not feel happy as to the prognosis of the case; in fact, to persist with it under such circumstances is a therapeutic error. The explanation of this retarding influence on the pulse is still a debatable point. By diminishing oxidation and retarding combustion it may have some effect, but the steadying of the pulse is out of proportion with the diminution of temperature. Possibly its effect on the vagus may in febrile conditions outweigh its effect on the accelerator nerves of the heart; and we must not forget a possible antiseptic effect upon the toxic products that already are urging on the heart to rapid action.

Again, in delirium, with the brain already in an intensely excited condition, it might be thought that to give alcohol under such circumstances was only adding fuel to the fire; but what is its effect? In delirium, combined with quick pulse and failing powers, we possess in alcohol a most important sedative, and in such conditions it must of necessity be prescribed. Even in *delirium tremens* in a vigorous man, we all know how much more certainly a dose of chloral will act if administered in a glass of bitter beer.

I do not wish it to be understood by these remarks that I would in *all* cases of fever give alcohol as a routine remedy. Many cases do quite well without it, in fact never want it; but what I do say is, that if nourishment be not taken, if the pulse shows the slightest inclination to abnormal rapidity and weakness, if there be the least indication of an idly moving tongue or the least suspicion of brain-weakness, then I say alcohol should not be withheld. I do not advocate waiting until the pulse is 140, the tongue is brown, and delirium only too manifest. Again a practical point: in a case of fever in a patient of middle life, or past that, I always think that previous habits should be taken into account. If to your knowledge that patient has been accustomed not necessarily to live freely, but only to take a moderate amount of alcohol as a daily beverage, then I should say by all means give that man alcohol from the first. He will need it sooner rather than later, and you had better be beforehand.

And now a few words on the influence of alcohol on the digestive tract. A robust and healthy man needs no alcohol either to sharpen his appetite or assist his digestion; but one whose vital energies are low either from being below par merely or convalescing from a severe illness, is all the better for a glass of wine. Nobody knows so well as a doctor how, after a long and arduous day at high pressure, the mere *thought* of food is distressing. He comes in weary both in body and mind; he flings himself down in a state of exhaustion; he is done up. He makes comments about "a dog's life," and all the world is wrong. Ask him if he is ready for dinner and hear what he says. But let him sit quietly in front of the fire and give him a glass of champagne, and what happens? He brightens up, he pulls himself together, changes his raiment and comes down to dinner with an appetite. The alcohol has done him good. Whether he is justified in repeating this sort of thing often is quite another story. But the contingency *will* arise sometimes, and this to my mind is the best way to meet it. To meet the feeble digestive power of the convalescent, a glass of generous port, or, if there be any tendency to nausea, a glass of champagne, is of great service.

In cases of colic and diarrhœa we all know how men prescribe for themselves port wine and brandy. The brandy acts as a sedative to the irritated nerves of stomach and intestines, whilst the astringent effect of the port is of service in allaying intestinal secretions. This occasionally suffices, but as often as not is useless, and it is as well to issue a word of warning on this point to our patients.

A man, after he has got to his business, is seized with an attack of "gripes," and immediately sends for and takes a stiff glass of brandy. He is perhaps temporarily relieved, but shortly he is as bad as ever. He then either comes round to see you or sends a messenger, and you find him in great pain, and, moreover, cold and miserable. The physiological action of alcohol has here been too much for him. The radiation of heat from his dilated skin capillaries has been sufficient to chill him severely; this, combined with the depressing effect of pain, has been too much for him, and you would do well to get him home and into a warm bed as quickly as possible. Had he taken the dose at home, in front of a good fire, he would probably have been spared all this, and the alcohol would have done him good.

The astringency of the red wines is well known, and in cases of irritable diarrhœa in middle-aged men, I have often found that a couple of glasses of red hock with luncheon have proved of great service.

The therapeutic value of alcohol in complaints of the genito-urinary system I will dismiss in a few words. We must be very cautious in our advice here. Gin with its juniper is known the wide world over as an excellent diuretic, and it is marvellous how inevitably it is taken when there is anything wrong imagined to be with "the kidneys." Even in our house-surgeon days we remember how every patient with retention of urine came in with his agony doubled after a liberal dose of gin "to make the water come." There are other diuretics to our hand, and I seldom, if ever, prescribe it for that purpose.

In women, who suffer from dysmenorrhœa and from pelvic pains, imaginary or real, the use of gin is unfortunately only too common, and we are skating over thin ice indeed in giving our sanction to its use. For my own part, I invariably discountenance it, and I have never regretted doing so.

It is not within the scope of my paper to wander off into the special action of alcohol in such general diseases as anæmia, chlorosis, diabetes, albuminuria, and so on, although there is much that is of vital interest to talk about in such matters; nor do I propose to even touch upon the pathological effects of chronic alcoholism.

One point with regard to anæsthetics. I never could see the rationale of mixing alcohol with chloroform and ether in the

A.C.E. mixture. Personally I never see any objection to giving a dessert-spoonful of brandy by the mouth before administration of an anæsthetic, just to give a little "Dutch courage," but from its volatility I doubt its good effects.

I must now hasten on, for time presses, to the discussion of alcohol as a beverage. To my mind, in the course of general practice, our advice on this point requires a *wise discretion.* Men who seek our counsel are more often than not possessors of the common-sense view on this matter, and woe betide our reputation if we are entrapped into a display of worldly ignorance. Once let a patient see that your knowledge of alcoholic drinks is shaky, and his confidence in you will experience a rude shock, and your control over his future alcoholic habits will be lost, for he will pursue his own course. That men *will* indulge in alcoholic drinks we must all of us admit, and the more we know of our subject the better shall we be able to guide them in that indulgence. A healthy man (and here I think we shall all agree) certainly does not require alcohol, but I am quite prepared to say that to partake of it as a luxury in moderate quantities is not fraught with any harm. Now moderation is a dangerous word, and has a different interpretation in various minds. For instance, we none of us could agree with the definition of moderation expressed by a young man who came to my consulting room the other day. I asked him how much drink he had taken in the course of the morning, and he replied, "Oh, nothing much!" but on being pressed, admitted that he had had seven glasses of whisky and two glasses of beer: and we could not allow ourselves to fall in with the view of the Scotchman as to moderation, who, after detailing the manner in which he disposed of thirteen glasses of whisky in the course of the day, said: "I canna understand a mon dram, dram, dramming all day long." No! the amount of alcohol that can be taken with impunity in twenty-four hours should be fixed; and we may safely agree with Parkes, who puts the limit at 1 to 1½ ounces of the absolute spirit in any given day. This corresponds to a sherry-glassful of brandy or whisky, to one pint of average beer, to two glasses of port or sherry, or to half a pint of claret. These data are trustworthy; and if a man will only adhere to these daily quantities he will come to no harm. If they be exceeded it should only be along with food at an occasional dinner party, that exquisitely refined debauch beginning with its glass of fortified sherry and ending with its cup of *café noir* as an antidote.

In recommending the particular drink that will suit an individual, we should certainly insist that the liquor be *good.* It is more important to recommend a wine merchant than to name a wine. In giving our advice we need not assume to ourselves the title of a *connoisseur;* but at any rate, we should be in a position to point out the relative values of different beverages under different circumstances. We should understand the relative values of red wines as compared with white, of effervescent wines as compared with still; we should know which beverages are most astringent and which most tonic; and we should have in our minds a clear knowledge of the meaning of such adjectives as *mature*, *fine*, *fruity*, *fortified*, *dry*, and *sweet*; and we should remember the rule that any liquor that retards the action of the kidneys must be avoided. In addition to these, we must count upon the factors of idiosyncrasy, hereditary failings, season of the year, present need, and length of purse.

There is as much dietetic folly in shabby gentility drinking bad claret as there is in a labourer drinking cheap gin. In each case the individual would fare far better on a glass of good wholesome British beer. Whatever beverage is recommended this rule is paramount—it should be good; and if the individual cannot afford that which is reliable, he is better without it altogether.

I should have liked to pursue in more detail the relative merits of the various alcoholic drinks, but time will not permit me.

To hold up to condemnation the immoderate use of alcohol, and to discuss the means whereby such abuse may be fought, is not our province to-night. I have only set myself the task of laying before you a few points for discussion of its *reasonable* usage. That alcohol is of use I have no doubt, and it is only by keeping ourselves abreast of the knowledge of our subject that we can guide our patients aright. If in the administration of our advice we are armed with this knowledge, and ever keep before our eyes the consciousness of the ultimate welfare of our patients, we may, by resolutely endeavouring to keep them within the limits we have prescribed, do more to stem the tide of intemperance than possibly we are aware.

---

